# Multiple large vessel aneurysmal formation in HIV-infected patients

**DOI:** 10.4102/sajr.v21i2.1186

**Published:** 2017-11-14

**Authors:** Ju-Mei Chang, Hassan Lameen, Garth C. Skinner

**Affiliations:** 1 Department of Radiology, Edendale Hospital, South Africa; 2 Department of Radiology, Nelson R. Mandela School of Medicine, University of KwaZulu-Natal, South Africa; 3 Department of ENT Surgery, Edendale Hospital, South Africa

## Abstract

A new form of aneurysmal dilatation of large vessels is becoming known in patients with human immunodeficiency virus (HIV) infection. We present a case report of a patient with this anomaly and discuss the radiological dilemmas involved in the diagnosis of the disease. This case highlights the need for computed tomography (CT) angiography as the imaging medium of choice.

## Introduction

Multiple mycotic aneurysm formation is an unusual disease usually associated with elderly atherosclerotic patients, posttraumatic victims, immunosuppressed patients or intravenous drug abusers. With the emergence of the HIV and/or AIDS pandemic, another bizarre form of vascular pathology is emerging. Multiple aneurysmal deformation of large vessels without an infective aetiology is being documented in HIV and/or AIDS patients with very low CD4 counts.^1^ There is an intracranial and extracranial presentation.

HIV-associated vasculopathy was first described in 1987, commonly presenting as arterial occlusive disease, aneurysmal disease or spontaneous arteriovenous fistula.^1^ The incidence of symptomatic vasculitis in HIV-infected patients is 1%.^1^

Imaging is necessary to establish the diagnosis, assess the number of aneurysms and characterise the aetiology of infective versus non-infective disease as well as mapping the anatomy for the surgeon to repair the defect. Computed tomography (CT) angiography is the current imaging modality of choice for any suspected arterial aneurysm, owing to its high resolution with 3D reconstruction, precise vascular anatomy and demonstration of possible associated complications which may influence the management planning. MR angiography is reserved for the patient who has limitations or contraindications for CT angiogram; with newer MRI techniques, there is improvement of the spatial and temporal resolution which allows us to competently evaluate the aorta, cerebral arteries and peripheral arteries.

Sonography can be of use if one suspects a peripheral arterial aneurysm in a patient, but generally it is unreliable in the diagnosis of aortic aneurysms.

## Case study

A 38-year-old man presented to a regional hospital with a 2-week history of right thigh pain radiating to his knee and lower back. He had recently been diagnosed with HIV with a CD4 count of 257 cells/µL. He had not started antiretroviral therapy. He had no previous history of trauma to the groin area. He was a smoker for the past 7 years. He was not diabetic or suffering from any concomitant cardiovascular disease.

On clinical examination, a palpable painful right inguinal mass was found. It was immobile and pulsatile, measuring 4 cm × 5 cm in size. There were no signs of overlying inflammation. His peripheral pulses were all present and equal in both legs. He walked with a limping gait.

His specialised investigations included a routine full blood count, urea and electrolytes. His white cell count of 8.6 × 10^3^ µL was normal. There were no other serum abnormalities detected.

**FIGURE 1:**
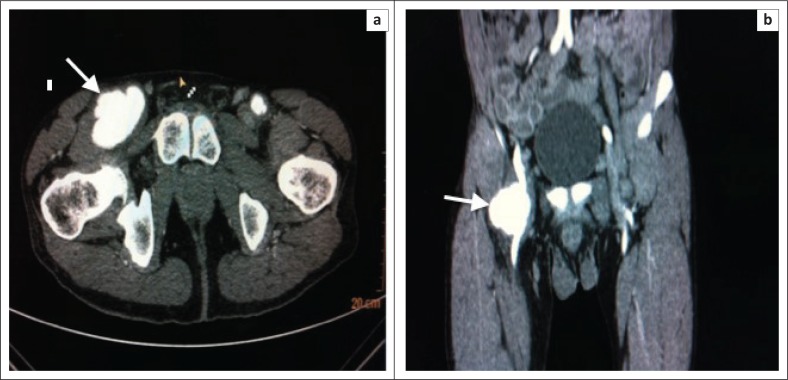
Contrasted axial and coronal images (a and b) demonstrate aneurysms seen at both inguinal regions (arrows), which are consistent with common femoral artery aneurysms. The right aneurysm measures approximately 5 cm in diameter, with significant surrounding perivascular oedema and enlarged inguinal lymph nodes. The left femoral aneurysm measures approximately 2 cm in diameter.

**FIGURE 2:**
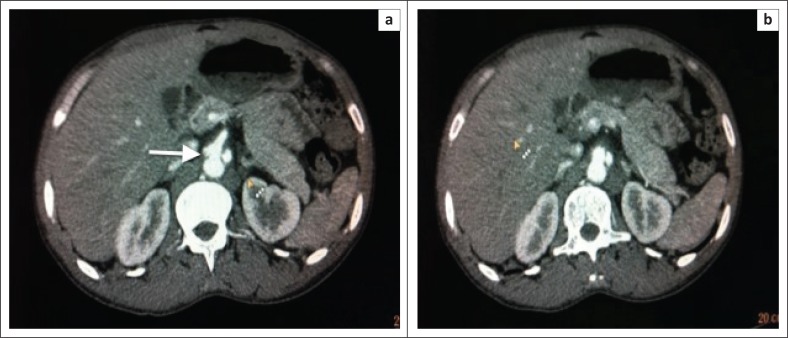
Axial contrast images (a and b) demonstrate dilatation of coeliac trunk with an outpouching representing another similar aneurysm. Perivascular soft tissue thickening may suggest an enlarged coeliac lymph node.

Initially, an ultrasound of his groin and abdomen was performed. The abdominal aorta was normal up to the level of the bifurcation. A right common femoral artery aneurysm was noted, measuring 4.26 cm in diameter. A second left sided common femoral artery aneurysm measuring 2 cm in diameter, with septation, was found. There was normal blood flow noted in the femoral arteries. Subsequently, a CT angiogram was performed. Bilateral common femoral artery aneurysms were present (Figure 1a and 1b). The larger right femoral aneurysm was associated with perivascular soft tissue oedema. There was dilatation of the coeliac trunk and a left sided abdominal aortic aneurysm (Figure 2a and 2b). As the patient lacked any clinical signs of being acutely ill, the diagnosis of multiple aneurysm formation in HIV infection was favoured rather than that of a multiple mycotic aneurysms. The patient was referred to a vascular unit at a tertiary hospital for surgical intervention.

## Discussion

Vascular complications, unique to HIV-infected patients, are now being seen due to the increasing incidence of HIV infection worldwide. They include multiple aneurysm formation, vasculitis and large vessel occlusive disease.^1^ It is believed that HIV-related multiple large vessel aneurysmal formation constitutes a distinct clinical and pathological entity.^2^ The aneurysms are usually multiple and saccular in nature, favouring the carotid and superficial femoral arteries. Other large vessel sites, for example, thoracic aorta, abdominal aorta, common iliac and popliteal, have also been regularly documented as being involved with aneurysmal formations. A multi-loculated appearance can be seen on imaging because of multiple false aneurysm formation along the vessel wall at the site of transmural necrosis.

The median age of presentation is between 30 and 40 years. This is much younger than patients presenting with atherosclerotic disease. Patients usually present with a subacute history of an expanding mass. Pain is the next most common symptom.^1^ This is very different to that of a patient presenting with a mycotic aneurysm. These patients are usually acutely ill, with multiple organ failure. However, a patient with HIV-related large vessel aneurysmal formation can present acutely because of haemodynamic instability from aneurysmal rupture or with respiratory distress because of airway compression.

These patients usually present at an advanced stage of immunosuppression. In Nair et al.’s series, 90% of their patients presented with a median CD4 count of < 400 cells/µL. Low serum albumin and elevated globulin levels were also consistent findings.^2^ They established that the inflammation affected the vasa vasora of the major vessels. An influx of neutrophils, plasma cells and lymphocytes causes an intense endothelial swelling which leads to a thrombotic occlusion of the vasa vasora. HIV proteins are noted within these lymphocytes, but the exact significance of this abnormality is yet to be defined. Transmural necrosis of the vessel wall occurs because of the probable ischemia and results in weakness and aneurysmal formation. The exact pathogenesis is still unknown. Theories such as direct virally mediated destruction or immune complex formation are favoured. There is slim supporting evidence that favours damage by another opportunistic pathogen such as mycobacterial, bacterial or syphilitic infections. Their series of 92 aneurysms in 28 patients yielded positive cultures in only 3 patients.^2,3^

The colour Doppler ultrasound features are typical of pseudo aneurysms with a defect or blow out of the vessel wall and turbulent pulsatile blood flow. There is also marked thickening of the vessel adjacent to the aneurysm and hyperechoic spotting of the arterial wall.^4^

The intracranial aneurysms described by Blignaut are usually single and at the anterior communicating artery. Most of the aneurysms have a neck width larger than 50% of the transverse width of the sac. Multiple aneurysms were more commonly found in the internal carotid artery.^5^

Du Pont et al, described the first case in Zimbabwe in 1989 that reported a Salmonella-related mycotic aneurysmal disease.^6^ Since then, increasingly more cases have been reported independent of bacterial infection. These cases show a predilection for young men who are in the advanced stages of HIV and/or AIDS as demonstrated by low CD4 levels.

Treatment of these aneurysms involves open exposure and repair, or endovascular therapy. The combination of a low CD4 count and low albumin levels (< 35 g/L) is a poor prognostic sign. Patients should be optimised prior to surgical repair.^7^

Bellows et al. have reported spontaneous regression of an abdominal aortic aneurysm in a HIV patient over a 6-month period. There are also other reports of mycotic aneurysms, although extremely rare, regressing on antibiotic therapy alone.^8,9^

## Conclusion

Relatively healthy HIV-infected patients presenting with a single, large vessel, arterial aneurysm must always be screened for other large aneurysms from the neck to the popliteal regions. The interesting finding in our case is the location of the aneurysms close to the main vessel bifurcations, a feature that demands further research. CT angiograms are the imaging modality of choice. In cases where CT imaging is contraindicated, Doppler ultrasound or MR angiography can be utilised. These cases should be prioritised because of the high nature of spontaneous rupture with immediate haemodynamic compromise.
